# Interfacial Pull-Out Properties of Surface-Grown Carbon Nanotubes (gCNTs) on Para-Aramid Fabric Material by Chemical Vapor Deposition (CVD)

**DOI:** 10.3390/nano15211637

**Published:** 2025-10-27

**Authors:** Erman Bilisik, Mahmut Korkmaz, Kadir Bilisik

**Affiliations:** 1 Nanotechnology Application and Research Centre (ERNAM), Erciyes University, 38039 Talas, Kayseri, Turkey; ermanbilisik66@gmail.com; 2 Department of Nanoscience and Nanotechnology, Graduate School of Natural and Applied Sciences, Erciyes University, 38039 Talas, Kayseri, Turkey; 3 Mustafa Cikrikcioglu MYO, Kayseri University, 38280 Talas, Kayseri, Turkey; korkmaz@kayseri.edu.tr; 4 Nano/Microfiber Preform Design and Composite Laboratory, Department of Textile Engineering, Faculty of Engineering, Erciyes University, 38039 Talas, Kayseri, Turkey

**Keywords:** CNT-grown aramid fiber, pull-out, fracture toughness, friction, intra-yarn shear

## Abstract

Carbon nanotubes (MWCNTs) were synthesized in situ on para-aramid fabrics (gCPO) via a low-temperature (450 °C) chemical vapor deposition (CVD) process to enhance interfacial pull-out, frictional, and fracture toughness characteristics. FESEM analysis confirmed CNT coverage on fiber surfaces, while FTIR, Raman, and XRD results indicated limited structural modification without significant polymer degradation. The CNT-functionalized fabrics exhibited a 66.19% increase in maximum pull-out force, 55.32% improvement in interlacement rupture strength, and a three-fold rise in intra-yarn shear resistance compared with control fabrics (KPO). The static and kinetic friction coefficients increased by 26.67% and 16.67%, respectively, due to CNT-induced surface roughness, enhancing inter-fiber load transfer and reducing slippage. Single-yarn pull-out tests revealed notable gains in energy dissipation and fracture toughness (up to 1769 J/m^2^), whereas multi-yarn pull-out performance decreased due to excessive friction surpassing filament strength. The study demonstrates that low-temperature MWCNT growth enables effective interfacial reinforcement of soft para-aramid fabrics, establishing a novel framework for meso-scale mechanical screening of flexible nano-ballistic composites.

## 1. Introduction

High-modulus para-aramid fibers enable the development of lightweight, flexible, and impact-resistant textile composites for applications such as aircraft fuselage reinforcements and personal armor systems. Ballistic performance of such structures is governed by fiber properties, weave topology, and frictional interactions at yarn interfaces [1,2,3]. Enhancing inter-yarn friction has been shown to increase energy absorption without compromising flexibility or areal density [4,5]. Various surface modification strategies—such as plasma treatment, chemical oxidation, sol–gel coating, and nanoparticle deposition—have been applied to improve interfacial bonding and frictional behavior in aramid systems [6,7,8,9]. However, high-temperature processes or aggressive chemical treatments often induce filament degradation. To address these limitations, nanomaterial-based approaches, particularly carbon nanotube (CNT) integration, have emerged as promising alternatives for enhancing inter-fiber adhesion and load transfer efficiency [10,11,12]. Hydrothermal growth of ZnO nanowires on p-aramid substrates improved inter-yarn friction, tensile strength, and stiffness, though with a moderate (~20%) mass increase [13]. Under impact, energy is dissipated through decrimping, axial elongation, lateral displacement, and yarn translation at crossover points [14]. Yarn pull-out and frictional sliding remain dominant mechanisms for impact energy absorption [15]. Additionally, strain rate effects influence failure morphology, with low-rate loading inducing extensive fibrillation, while high-rate impacts lead to localized and brittle fibril fracture in aramid filaments [16].

Yarn-to-yarn friction in hybrid structures has been quantified using twisted strand and capstan methods. These metrics, evaluated under variable tension, speed, angle, and temperature, are essential for modeling fabric shear behavior and formability [17,18,19,20,21,22]. Frictional assessments of aramid and ultra-high-molecular-weight polyethylene (UHMWPE) yarns using the slope method confirmed higher coefficients for aramid, though both materials exhibited static friction values below 0.3, indicating room for improvement in ballistic contexts [23]. Tow-level friction, inherently anisotropic due to surface texture and filament alignment, plays a critical role in high-performance yarn processing. Capstan tests on aramid, carbon, and e-glass tows under varying contact modes revealed surface topography and tow orientation as dominant factors [24]. Surface fibrillation methods yielded up to sevenfold increases in pull-out energy and sixfold gains in peak force [25]. Moreover, frictional behavior is influenced by yarn texture, surface treatments, and environmental conditions. Hydrophilic surfaces showed humidity-dependent friction and hydrophobic ones remained stable [26]. Nanostructured coatings (e.g., titanium dioxide/zinc oxide (TiO_2_/ZnO), silicon dioxide (SiO_2_), silver nanorods (AgNRs)) enhanced inter-yarn friction, strain energy, and ballistic resistance with negligible mass gain. Silica coatings and AgNRs notably improved pull-out resistance and induced anisotropic frictional responses [27,28,29,30,31,32,33].

The shear response of p-aramid fabrics was characterized using the yarn pull-out technique, which effectively captures inter-yarn friction and structural interactions. Shear strength and rigidity were found to increase with fabric density, specimen size, and number of extracted yarns, while shear jamming angles exhibited positive correlation with pulled-end count [34,35]. Notably, width-to-length ratio also influenced shear parameters, validating pull-out testing as a reliable method for evaluating in-plane shear performance. Interlacing point resistance governs yarn mobility and structural cohesion, making it a critical factor in woven fabric mechanics. Complementary bias extension tests—sensitive to yarn orientation and fabric architecture—further elucidate non-linear deformation patterns under shear [36,37]. Shear deformation progresses through successive stages: micro-friction at interlacement points, rotational displacement of tows, and progressive locking with increased contact pressure, leading to localized bending, buckling, and wrinkling [38]. These mechanisms facilitate fabric drapability into complex forms [39]. The onset of wrinkling coincides with peak shear angles, demarcating the fabric’s in-plane deformation limit and internal yarn frictional resistance [40].

Aramid fiber composites are often limited to ballistic applications due to weak fiber–matrix adhesion, delamination risks, and poor multifunctionality. To mitigate interfacial limitations, laser-induced graphene (LIG) has been directly synthesized on aramid fabrics, enabling tunable surface architectures that enhance mechanical interlocking and interlaminar performance [41]. Similarly, metallic coatings (copper (Cu), aluminum (Al), aluminum nitride (AlN), silver (Ag)) via directed vapor deposition increased inter-yarn friction in p-aramid yarns, though with reduced tensile integrity [42]. Chemical vapor deposition (CVD) offers a versatile, scalable platform for in situ CNT synthesis, enabling direct integration on fiber surfaces with moderate control over nanotube alignment and density [43,44]. CNT growth on carbon fibers has shown varying effects: enhanced mechanical interlocking and dielectric properties [45,46], modest increases in interlaminar/interfacial shear strength (ILSS/IFSS), and, in some cases, decreased IFSS due to poor CNT–epoxy bonding and stress concentrations [47]. Subsequent modifications—such as heat treatment, silane functionalization, or silica coating—have improved stress transfer, fracture toughness, and conductivity [48,49,50,51,52,53,54,55]. Various surface modification techniques have been explored to improve the interfacial bonding of para-aramid fibers with polymer matrices. Conventional approaches such as plasma treatment, chemical oxidation, sol–gel coating, and high-temperature CVD (>650 °C) have demonstrated partial improvements in fiber–matrix adhesion, surface roughness, and inter-yarn friction. Plasma and oxidative treatments typically enhance surface activity and wettability but provide only limited and short-term interfacial stability. Sol–gel and metallic oxide coatings introduce nano-scale roughness and improved chemical affinity, though they often form brittle or poorly adherent interlayers. Conventional high-temperature CVD processes enable CNT deposition on fiber surfaces but tend to degrade the thermal and mechanical integrity of the para-aramid substrate. Chemical vapor deposition (CVD) provides a scalable method for in situ CNT growth on fiber surfaces, yet conventional high-temperature (>650 °C) CVD treatments cause thermal damage to para-aramid polymers. Recent studies have shown that the pressure and thermodynamic environment during CVD play a decisive role in controlling defect density, alignment, and crystallinity of the grown nanostructures. It was reported that low-pressure epitaxial CVD enables precise interfacial registry and lattice coherence, leading to defect-free nanostructures with enhanced interfacial bonding [56]. Similarly, it was demonstrated that varying CVD pressure from 1 Torr to 760 Torr significantly alters vacancy formation and chemical passivation behavior, where atmospheric pressure promotes defect healing through oxygen incorporation [57]. Recent advances in CVD methodologies have also underscored the process’s tunability and scalability for the controlled synthesis of graphitic nanostructures [58].

In contrast to previous studies that primarily focused on high-temperature CNT growth on carbon or glass fibers, the present work introduces a low-temperature (450 °C) in situ CVD process enabling the stable growth of multi-walled carbon nanotubes (MWCNTs) directly on soft para-aramid fabrics without significant polymer degradation. This unique approach allows the systematic investigation of single- and multi-yarn nano-pull-out behavior, interfacial friction, and fracture toughness, providing quantitative insight into meso-scale energy dissipation mechanisms in flexible fiber assemblies. Therefore, the objective of this study is to develop a low-temperature (450 °C) in situ CVD-assisted MWCNT growth technique for para-aramid fabrics and to systematically characterize its influence on nano pull-out, interfacial friction, and fracture toughness. This work introduces a novel meso-scale mechanical pre-screening framework for evaluating the interfacial and energy absorption performance of flexible nano-ballistic composites prior to full-scale testing.

## 2. Experiment

### 2.1. Materials

**Figure 1:**
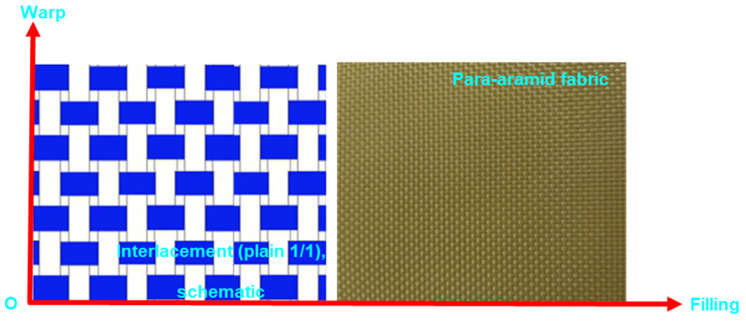
Schematic illustration followed by the corresponding view of the plain-weave (1/1) para-aramid fabric.

The woven fabric was manufactured from para-aramid fibers supplied by Teijin Inc. (Tokyo, Japan). Both warp and weft yarns exhibited a linear density of 336 tex. The fabric corresponds to the Twaron CT^®^ 747 model (Öztek Tekstil Terbiye Tesisleri San. ve Tic. A.Ş., Tekirdağ, Turkey) and features a plain-weave (1/1) architecture. The yarn densities in the warp and weft directions were determined to be 6.25 ends/cm. The areal density of the fabric was measured as 410 g/m^2^. The crimp percentages for warp and weft yarns were identified as 5.80% and 5.90%, respectively. The fabric exhibited a thickness of 0.62 mm and had been subjected to a water-repellent surface treatment. Figure 1 presents both the actual image and schematic representation of the fabric structure. Furthermore, the para-aramid fiber exhibits a diameter of 12 μm, a density of 1.45 g cm^−3^, a tensile strength of 3200 MPa, a tensile modulus of 115 GPa, and an elongation-at-break of 2.9%, with a thermal degradation onset temperature of approximately 450 °C [59].

### 2.2. Fabrication of Para-Aramid Substrates Through Chemical Vapor Deposition (CVD) Processing

**Figure 2:**
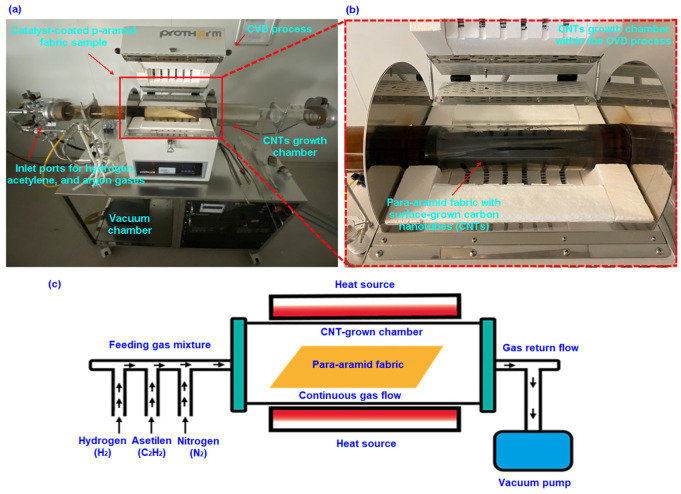
Surface growth of carbon nanotubes (CNTs) on p-aramid fabric via chemical vapor deposition (CVD). (**a**) The furnace process employed for CVD synthesis, (**b**) the localized CNT growth zone within the CVD reactor chamber, and (**c**) a schematic representation of the CVD growth system (digital images).

**Figure 3:**
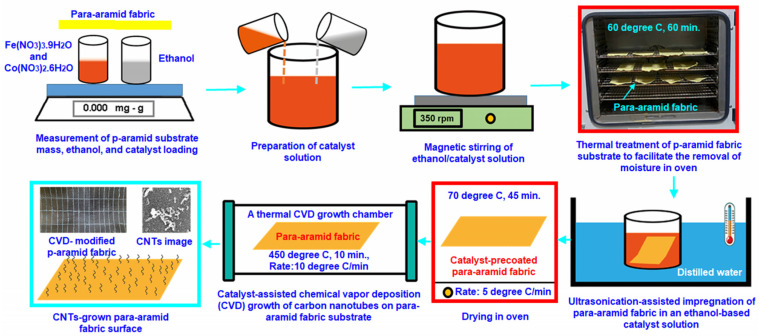
Process flow schematic depicting the sequential stages of carbon nanotube (CNT) growth on para-aramid fabric through a chemical vapor deposition (CVD) route.

Table 1 presents carbon nanotube (CNT)-grown para-aramid fabric surface tailored for yarn pull-out performance evaluation. Moreover, Figure 2a–c illustrates the application of the chemical vapor deposition (CVD) process onto the para-aramid fabric. Additionally, Figure 3 illustrates the stepwise schematic of the carbon nanotube (CNT) synthesis procedure on p-aramid fabric substrates via the CVD process.

The synthesis of CNTs (multi-walled carbon nanotubes, MWCNTs) on para-aramid fabric (gCPO) via the CVD technique was conducted at the Nanoscience and Nanotechnology Research Center (ERNAM) of Erciyes University, located in Talas, Kayseri. The following procedure outlines the steps implemented to achieve CNT growth on the para-aramid substrate at relatively low temperatures to preserve the fabric’s structural integrity. Initially, para-aramid fabric samples were cut into 10 cm × 30 cm strips, and the water-repellent surface finishes were removed. This was accomplished by heating the fabric at 60 °C for 60 min at heat rate of 15°/min. Subsequently, the catalyst solution was prepared by dissolving iron (III) nitrate nonahydrate (Fe(NO_3_)_3_·9H_2_O) and cobalt (II) nitrate hexahydrate (Co(NO_3_)_2_·6H_2_O) in ethanol at a 1:1 molar ratio, yielding a final concentration of 0.05 mol/L. The mixture was magnetically stirred at 350 rpm for 10 min, followed by an additional 10 min of ultrasonication. The chemically cleaned para-aramid fabric was then immersed in the catalyst solution under ultrasonic agitation for 10 min to ensure uniform catalyst deposition. After coating, the samples were dried at 70 °C for 45 min at heat rate 5°/min to form a stable catalyst layer on the fiber surface. Once dried, the fabric was centrally positioned in the CVD furnace chamber (Protherm furnaces, STF series, max. temp. 1200–1300 °C, heated length 600 mm, heated diameter 100 mm, 5 kW, TR). The system was evacuated, and the reactor pressure was adjusted to 750 Torr (0.099 MPa). The furnace temperature was elevated to 450 °C at a ramp rate of 10 °C/min under continuous nitrogen gas flow (0.3 L/min). This target temperature was determined based on thermogravimetric analysis (TGA) data of the fabric, corresponding to the point at which mass loss was minimized. Upon reaching the desired temperature, a gas mixture composed of hydrogen (H_2_), acetylene (C_2_H_2_), and nitrogen (N_2_) was introduced to initiate carbon nanotube (CNT) growth. The mixture was fed into the system at controlled flow rates of 0.15/0.15/0.3 L/min, respectively, and the reaction was sustained for 10 min. Following the CNT synthesis stage, the flows of hydrogen and acetylene were terminated, and the system was allowed to cool down to 25 °C under continuous nitrogen flow. These process parameters comprehensively define the CNT growth procedure on para-aramid fabric substrates. These process parameters define the in situ growth of MWCNTs on para-aramid fabric substrates. Moreover, the weight and volume fraction results of the synthesized multi-walled carbon nanotubes are summarized in Table 2.

The selection of 450 °C as the CNT growth temperature was guided by the TGA/DTA results of the p-aramid fabric, which indicated only about 1% weight loss at this temperature. Although individual fibers extracted from the CNT-grown fabrics exhibited a significant reduction in tensile strength compared with the control (KPO), this behavior reflects a fundamental trade-off between maintaining polymer integrity and achieving sufficient catalytic activity for CNT nucleation. At lower temperatures, CNT growth could not be initiated, whereas higher temperatures caused severe structural relaxation. Hence, 450 °C was identified as the optimum condition enabling catalytic activation of Fe/Co nanoparticles and acetylene decomposition while preventing extensive degradation of the p-aramid substrate. FTIR and Raman analyses confirmed the retention of characteristic amide (C=O, C–N) and aromatic (C=C) bands, and FESEM micrographs revealed that the fiber surface morphology remained intact. This controlled thermal exposure permitted continuous, adherent MWCNT networks to form, providing effective interfacial load transfer and energy dissipation in the composite system. The modest loss in intrinsic fiber strength is therefore considered an acceptable engineering compromise that allows stable CNT anchoring and improved inter-filament performance in flexible nano-ballistic fabrics.

The weight-to-volume fraction formula and calculation of the grown CNTs were derived based on the following correlations (1)–(4).

(1) Wvf=Wgfeff−WlossWgfeff×100(%)

(2) $$W_{loss}=0.0962426\times W_{d}$$

(3) $$W_{gfeff}=W_{gf}-W_{ct}$$

(4) $$W_{ct}=W_{cf}-W_{df}$$

where *W_d_* is the mass of the control fabric (g); *W_df_* is the mass of the fabric after chemical removal treatment (g); *W_cf_* is the mass of the catalyst-deposited fabric (g); *W_gf_* is the mass of the catalyst-deposited fabric after carbon nanotube (CNT) growth (g); *W_loss_* is the residual mass of the fabric at 450 °C (g); *W_gfeff_* is the net mass of the synthesized multi-walled carbon nanotubes (g); *W_vf_* is the weight percentage of the grown CNTs (%); *W_ct_* is the net catalyst mass deposited on the fabric (g); and 96.2426% is the residual mass fraction of the control para-aramid fabric at 450 °C as obtained from the TGA/DTA thermogram.

The net CNT content on the para-aramid substrate was 0.315 wt.% (Table 2). Although small on a mass basis, this value is typical for surface-grafted CNT architectures on polymeric fibers and reflects a thin, conformal nanotube network on the outer filament surface. Such sub-percent loadings are probably sufficient to modify inter-fiber friction and load transfer while avoiding excessive mass addition and thermal damage. In this study, the CVD parameters (450 °C, 10 min, 750 Torr) were tuned to prioritize interfacial reinforcement with minimal polymer degradation, rather than maximizing CNT mass.

### 2.3. Analysis and Characterizations

Morphological, structural, and chemical characterizations were comprehensively performed to evaluate the effects of in situ carbon nanotube (CNT) growth on the para-aramid fabric substrates. The objective was to correlate the nanoscale surface alterations with corresponding mechanical, thermal, and interfacial property modifications. All analyses were conducted at the Nanotechnology Application and Research Center (ERNAM), Erciyes University, under controlled environmental conditions (22 ± 1 °C, 45 ± 5% RH).

#### 2.3.1. Field Emission Scanning Electron Microscopy (FESEM) and Energy Dispersive X-Ray (EDX) Analysis

The surface morphology, topographical uniformity, and CNT distribution on the para-aramid fabric were investigated using field emission scanning electron microscopy (FESEM; ZEISS GeminiSEM500, Oberkochen, Baden-Württemberg, Germany) at accelerating voltages of 3–10 kV. Both low- and high-magnification images (up to ×200,000) were captured to evaluate the degree of CNT nucleation, filament coverage, and inter-filament continuity. Prior to imaging, specimens were mounted on aluminum stubs using carbon tape and sputter-coated with a gold layer to enhance surface conductivity. Elemental composition and spatial distribution of catalyst residues (Fe and Co) as well as carbon presence were confirmed via energy-dispersive X-Ray spectroscopy (EDX) integrated within the FESEM system. Prior to the yarn pull-out experiments, low-magnification (6.7×) optical micrographs depicting the yarn configuration were captured using an Olympus SZ61 stereo microscope equipped with Bs200DOC digital imaging software (Olympus Corporation, Hachioji, Tokyo, Japan). Additionally, macro-scale visual documentation of specimen configurations and testing procedures was acquired with a Nikon D3000 digital SLR camera (10.2 MP; 18–55 mm AF-S DX VR lens, Nikon Corp., Tokyo, Japan).

#### 2.3.2. FTIR, Raman, XRD, and TGA Characterizations

Surface chemical alterations resulting from the CNT growth process were characterized using Fourier-transform infrared spectroscopy (FTIR; Spectrum 400, PerkinElmer^®^, Shelton, CT, USA) in absorbance mode across the 4000–400 cm^−1^ range with a spectral resolution of 2 cm^−1^. This analysis was to monitor structural modifications after CVD treatment. Comparative analysis between pristine (KPO) and CNT-functionalized (gCPO) fabrics was conducted to detect shifts or intensity variations in the related regions. These spectral differences were used as indicators of potential CNT–aramid interfacial interactions.

Raman spectroscopy (alpha300, WITec GmbH, Ulm, Germany) employing a 532 nm laser excitation was used to further identify vibrational changes associated with CNT growth and to validate the presence of characteristic CNT bands. Simultaneously, the Raman spectra of the para-aramid structure were assessed to evaluate the stability of the polymer backbone and the extent of CNT–polymer coupling. The detection of CNT-related Raman features confirmed the successful deposition of CNTs on the aramid surface and provided complementary insights into the microstructural interactions observed via FESEM.

Crystalline structure and phase transitions induced by the CVD process were examined using X-Ray diffraction (XRD; Empyrean, Malvern Panalytical, Malvern, Worcestershire, UK) operating with Cu Kα radiation (λ = 1.5406 Å) in the 2θ range of 0–40°. The diffraction patterns were used to distinguish the intrinsic crystalline peaks of para-aramid filaments from newly formed carbonaceous phases attributed to CNT growth. Comparative analysis between untreated and CNT-grown specimens allowed for the identification of structural reorganization, revealing how CNT deposition influenced the overall structural integrity of the fiber substrate.

The thermogravimetric and differential thermal analyses (TGA/DTA; Hitachi STA 7300, Hitachi High-Tech Corporation, Tokyo, Japan) were performed to determine the thermo-oxidative stability and degradation behavior of the CNT-grown and pristine para-aramid fabrics. Samples weighing approximately 3–4 mg were heated in alumina crucibles from 25 °C to 1000 °C at a rate of 10 °C/min under a nitrogen flow of 1.5 mL/min. The weight loss profiles and derivative curves (DTG) were examined to identify the onset and peak decomposition temperatures. The data were used to establish a correlation between CNT synthesis temperature (450 °C) and the thermal degradation limits of para-aramid fibers, thus validating the thermal process parameters adopted during CVD.

The combined data from FESEM–EDX, FTIR, Raman, XRD, and TGA analyses provided an integrated understanding of the CNT–aramid interfacial system. Morphological evidence from FESEM and compositional confirmation from EDX established the physical presence of CNTs, while FTIR and Raman analyses confirmed chemical interactions and vibrational alterations within the aramid backbone. XRD and TGA data further substantiated the thermally induced structural modifications and their implications on mechanical behavior. Collectively, these characterizations validated the successful nanoscale functionalization of para-aramid fabrics via the CVD route and elucidated the microstructural mechanisms governing the enhanced pull-out and interfacial performance observed in subsequent tests.

#### 2.3.3. Nano Pull-Out Testing

Yarn pull-out experiments were conducted to quantify inter-yarn frictional behavior in both single- (1-end) and multiple- (2- and 3-end) yarn configurations along the frayed edges of plain weave (1/1) para-aramid fabrics. Tests were performed using a custom-engineered fixture [60] mounted on a universal testing machine (Instron 4411, 5 kN load cell, Instron Corp., Norwood, MA, USA). Nano-functionalized fabric specimens (50 mm × 200 mm) were prepared with a controlled fray length of 100 mm and a total clamped edge length of 700 mm, as illustrated in Figure 2a–c. Individual yarns were sequentially extracted (1st, 2nd, and 3rd), followed by dual- and triple-yarn extractions to simulate multiple-yarn frictional interactions.

All tests were executed in the warp direction under negligible pre-tension at a constant crosshead speed of 100 mm/min. The corresponding force–displacement (F–δ) profiles were recorded to determine peak pull-out loads, crimp extension (i.e., tensile straightening of yarn undulation), onset of interlacement failure, and characteristic stick–slip responses. Crimp extension was defined as the elongation attributable to structural waviness of the yarns embedded in the weave [60]. Displacements preceding crimp removal and localized yarn rearrangements were disregarded due to constrained boundary conditions. For consistency, warp/weft slippages and intra-yarn filament ruptures were omitted from the analysis. Measurement alignment was facilitated using 10 mm pick-spacing markers along the sample axis.

#### 2.3.4. Nano Yarn Tensile Strength Testing

Tensile performance of CNT-grown yarns (warp), extracted from para-aramid fabric specimens (120 mm width ×300 mm length), was assessed to determine the influence of nano-functionalization on yarn-level mechanical integrity. Testing was conducted in accordance with the TS EN ISO 2062 standard [61], utilizing a universal testing machine (Instron 4411, Instron Corp., Norwood, MA, USA) at a constant crosshead speed of 150 mm/min. The tested yarns possessed a linear density of 336 tex and a gauge length of 150 mm. Crimp-induced effects were excluded from the analysis, as the extracted nano-treated yarns retained a straightened geometry with no entanglement, and nanoparticles appeared uniformly distributed along filament surfaces. The yarn strength (σ_y_) was computed using Equation (5):

(5) $$\sigma_{y}=\frac{F_{max}}{W_{y}}$$

where *σ_y_* denoted the yarn tensile strength (N/tex, tenacity), *F_max_* is the maximum tensile load (N), and *W_y_* represents the yarn linear density (tex).

#### 2.3.5. Intra-Yarn Shear Strength

The intra-yarn shear strength (IYSS) was investigated based on the mechanical interaction of single and multiple warp yarns subjected to axial pull-out along the warp direction. During extraction, angular distortion of the filling yarns within the fabric plane occurred prior to the manifestation of stick–slip behavior. This phenomenon is critical for assessing shear resistance at warp-to-warp and warp-to-filling yarn interfaces during the pull-out process. Accordingly, IYSS of nano-functionalized para-aramid fabrics was calculated using Equation (6):

(6) $$\tau_{iy}\left(IYSS\right)=\frac{F_{max}}{\pi Rl_{y}}$$

where *τ_iy_* is the intra-yarn shear strength (N/m^2^, Pa), *F_max_* denotes the maximum pull-out force (N), *R* is the yarn diameter (mm), and *l_y_* represents the yarn length within the fabric (mm).

The analysis was conducted under the assumptions that fabric crimp and in-plane wrinkling effects were negligible, and that the yarns behaved as idealized, inextensible multifilament structures. Post-processing and computation of IYSS, along with nano pull-out and yarn tensile strength data, were performed using Microsoft Excel 2016.

#### 2.3.6. Pull-Out Energy Analysis

The pull-out energy (J) corresponding to single- and multi-end yarn extractions was determined by calculating the area under the load–displacement (F–δ) curves, thereby quantifying the energy dissipated during crimp extension, onset of interlacement failure, and subsequent stick–slip phases. The total energy absorbed was computed as the cumulative integral across all deformation regimes.

Numerical integration of the F–δ data for all nano-functionalized fabric configurations was performed using Python (v3.1.2.4, The Python Software Foundation, Amsterdam, The Netherlands), leveraging its scientific computing libraries to ensure precision and reproducibility in energy calculations.

#### 2.3.7. Pull-Out Fracture Toughness

**Figure 4:**
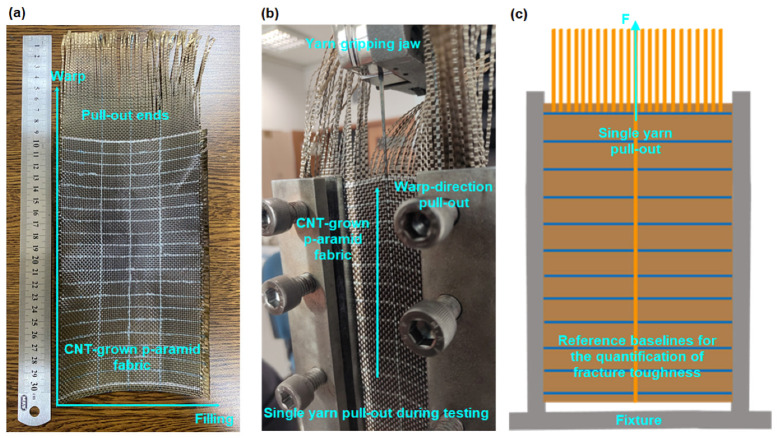
Pull-out testing configuration for CVD-treated p-aramid fabric. (**a**) A specimen of carbon nanotube (CNT)-grown para-aramid fabric, (**b**) a real-time image of CNT-grown fabric undergoing pull-out testing, and (**c**) a schematic representation of the test setup and gripping fixture (digital images).

Pull-out fracture toughness was quantified as the energy dissipated per unit area during yarn extraction, specifically within the stick–slip domain of the force–displacement (F–δ) profile. This parameter characterizes the resistance imparted by warp–weft interlacement to yarn mobility within the gCPO fabric. To enable spatially resolved analysis, fabric samples were pre-marked at uniform intervals along the extraction axis according to pick density (e.g., 10 mm spacing), as illustrated in Figure 4a–c. Upon reaching the peak extraction force, the corresponding yarn displacements at each designated interval were calculated by summing crimp extension values extracted from the F–δ curves. Pull-out fracture toughness (G_pf_, J/m^2^) was computed in accordance with the mode-I fracture energy principles outlined in ASTM D5528-01, using Equation (7) [62,63]:

(7) $$G_{pf}=\frac{F_{i}\times \delta_{i}}{b\times a_{i}}$$

where *G_pf_* is the pull-out fracture toughness (J/m^2^), *F_i_* denotes the force at a given displacement increment (N), *δ_i_* is the corresponding displacement including crimp extension (mm), *b* is the specimen width (mm), and *a_i_* represents the effective yarn raveling length (mm). Yarn axial elongation was considered negligible, and only crimp extension was accounted for in deformation analysis. The extracted yarns were modeled as inextensible multifilament structures. All post-processing and calculations related to pull-out energy, interfacial friction, and fracture resistance were performed using Microsoft Excel (v2016).

#### 2.3.8. Nano Friction Testing

**Figure 5:**
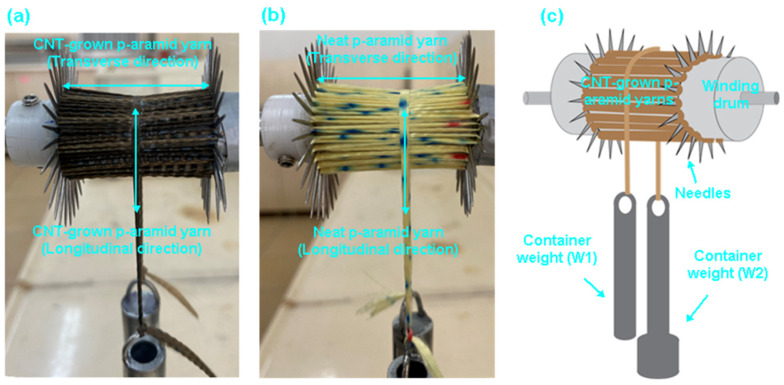
Frictional testing for CVD processed fabric substrate. (**a**) CNT-grown p-aramid yarn, (**b**) control p-aramid yarn, and (**c**) schematic representation of in-house-designed frictional instrument (digital image).

The frictional response of CNT-grown p-aramid yarns was investigated under both dry and wet conditions through static friction testing of single- (1-end) and multi-end (2- and 3-end) yarn configurations, employing a custom-built capstan-based test apparatus [64]. Prior to the wet friction tests, the CNT-grown para-aramid yarns were lightly sprayed with distilled water until no visible droplets remained on the surface. The samples were then conditioned for 1 h at standard laboratory conditions (25 ± 2 °C, 50 ± 5% RH) to ensure uniform surface moisture distribution. After the conditioning period, the wet friction tests were performed under the same environmental conditions. This procedure allowed consistent moisture absorption in the fiber surfaces without inducing surface pooling or slippage effects. As depicted in Figure 5a–c, the test setup consisted of friction drums mounted on a galvanized steel frame (450 × 60 × 30 mm), secured onto a metallic base (250 × 200 × 10 mm). Each drum incorporated a central galvanized steel core (10 × 130 mm) encased within a polyoxymethylene outer sleeve (20 × 70 mm), surrounded by peripheral guide needles (1 × 15 mm) to facilitate controlled cross-winding of yarns.

In the test procedure, a 336-tex nano-functionalized warp yarn was helically wound in a crosswise pattern around the drum. A secondary 336-tex yarn segment (150 mm in length) was positioned longitudinally anchored at one end to a constant load (W_1_ = 120 g) and connected at the opposite end to a variable load (W_2_) suspended via an iron-powder-filled container. Frictional force was quantified by incrementally increasing W_2_ until incipient motion occurred, at which point the corresponding mass was recorded. For multi-yarn configurations, yarns were arranged in parallel without overlap. Yarn extensibility and crimp-induced effects were disregarded, and a CNT-grown with non-entangled filament morphology was assumed. The static coefficient of friction (μsc) was calculated based on classical capstan mechanics (Euler equation) using the following expression (8) [50,65]:

(8) $${µ}_{sc}=\frac{1}{\pi }\ln\frac{W_{2}}{W_{1}}$$

where *W*_1_ is the stationary weight (g), *W*_2_ is the variable weight (g), and *µ_sc_* is the coefficient of static friction.

The kinetic friction coefficient (μₖₚ) was extracted from the post-interlacement stick–slip zone of the force–displacement (F–δ) curves. This value was calculated at regular 10 mm pick intervals, and the mean of the dataset was used to represent the dynamic frictional behavior of the CNT-grown fabric.

#### 2.3.9. Fabric Specification Tests

Fabric crimp was evaluated using a Tautex digital crimp tester (James H. Heal Co., Halifax, UK), following the TS 254 ISO 7211-3 standard protocol [66]. Thickness measurements were carried out with a calibrated cloth thickness gauge (R&B model, James H. Heal Co., Halifax, UK) in accordance with TS 7128 EN ISO 5084 [67]. Areal density (fabric mass per unit area) was determined according to TS 251 (ISO 3801) [68].

## 3. Results and Discussion

### 3.1. Morphological, Chemical, and Structural Results

#### 3.1.1. FESEM Results

**Figure 6:**
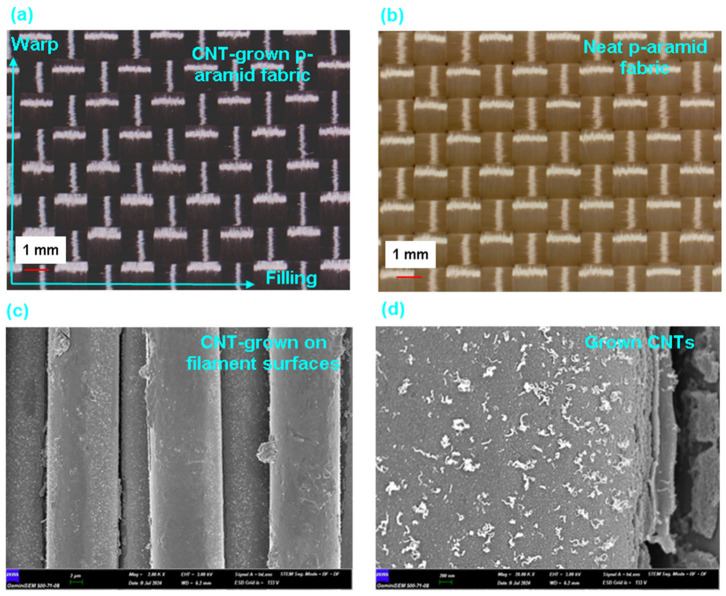
Field emission scanning electron microscopy (FESEM) images illustrating the surface morphologies of p-aramid fabric substrates subjected to chemical vapor deposition (CVD) processing. (**a**) Surface of CNT-grown p-aramid fabric (optical microscopy, ×6.7), (**b**) pristine p-aramid fabric surface (optical microscopy, ×6.7), (**c**) carbon nanotubes (CNTs) synthesized on individual filament surfaces (×2 μm) and (**d**) magnified view of CNTs grown on the filament (×200 nm).

Figure 6a–d presents field emission scanning electron microscopy (FESEM) images illustrating the surface morphologies of para-aramid fabric substrates subjected to chemical vapor deposition (CVD) processing. As can be observed in Figure 6a,b, the CVD-induced growth of carbon nanotubes (CNTs) on the p-aramid fabric led to a noticeable color transformation from its original yellow to a darker brown tone with particle blackened regions. According to the FESEM observations, CNT growth was particularly dense in the intra-filament regions compared to other surface areas (Figure 6c). A closer examination of the filament surface reveals that the CNTs were grown in a curved morphology with relatively short lengths (~400–700 nm) and thick tubular diameters (~25–50 nm) (Figure 6d). As discussed in the force–displacement behavior section (pull-out performance), CNT growth at elevated temperatures (450 °C) significantly deteriorated the internal structure of the para-aramid filaments in which their amide bonds are probably susceptible to thermal cleavage, resulting in a dramatic reduction in their tensile strength (over 5 times). Therefore, it is recommended that CNT growth via CVD be conducted at lower processing temperatures to preserve fiber integrity, although further investigation is warranted to optimize process parameters.

The EDX spectra of CNT-grown para-aramid fibers revealed distinct Fe and Co signals, confirming the presence of residual catalyst nanoparticles distributed across the fiber surface. During CVD treatment, the initial Fe/Co nitrate precursors were thermally decomposed and partially reduced under H_2_/C_2_H_2_/N_2_ atmosphere, forming Fe–Co metallic or metal oxide nanoparticles that served as nucleation sites for CNT growth. These catalyst residues were uniformly dispersed without forming large agglomerates, as evidenced by localized contrast variations in the FESEM micrographs. The resulting catalyst state is probably expected to consist of a mixed Fe^0^, Co^0^ (metallic) and FeO, CoO (metal oxide) phase [44,51], which is typical for CNT growth at 450 °C.

Although surface roughness was not evaluated in this study, this limitation arises mainly from the highly crimped and interlaced nature of the woven para-aramid fabric, which prevents accurate nanoscale surface height measurement. The complex topography of the yarn intersections and macro-scale undulations would dominate the roughness readings and mask the true effect of CNT coating. Therefore, surface morphology evaluation was focused on FESEM observations, which provided clear and direct evidence of MWCNTs across the fiber surfaces. Future work may focus on performing roughness mapping and surface analysis on flattened or nano particle incorporated para-aramid fibers to quantitatively correlate the nanotube-induced topographical changes and surface energy alterations observed after low-temperature CVD treatment.

Although the FESEM images in Figure 6 clearly demonstrate CNT growth across the para-aramid fiber surface, no statistical areal density quantification was performed in this study. Given the presence of thousands of individual filaments within the yarn TOW, extracting reliable CNT density values for each filament would require advanced image analysis and high-resolution mapping beyond the current experimental capacity. Future work will focus on establishing a correlation between CNT areal density, surface coverage ratio, and interfacial load transfer mechanisms using automated image-processing techniques.

#### 3.1.2. FTIR Spectroscopy Results

**Figure 7:**
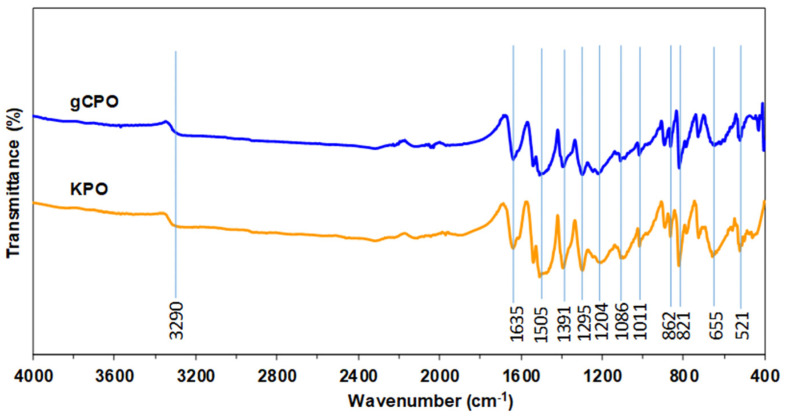
Fourier-transform infrared (FTIR) spectroscopy data of CNT-grown p-aramid substrate and neat p-aramid fabric.

Figure 7 illustrates the FTIR spectroscopy data of CNT-grown p-aramid substrate and neat p-aramid fabric. A broad absorption band around 3290 cm^−1^, characteristic of N–H stretching vibrations in amide groups, is clearly observed in both samples. However, the intensity of this band is reduced in the gCPO spectrum, suggesting that high-temperature exposure during CNT growth probably led to partial degradation of amide groups [69]. Moreover, the prominent peaks at 1635 cm^−1^ and 1505 cm^−1^ correspond to C=O stretching (amide I) and N–H bending (amide II), respectively. While these peaks are sharp in both spectra, a relative decrease in intensity in the gCPO sample further supports the thermal weakening of amide linkages due to the CVD process. In the region between 1391 cm^−1^ and 1011 cm^−1^, which includes C–N stretching and aromatic ring vibrations, a slight broadening or shift is observed in the gCPO spectrum. These shifts could be attributed to structural reorganization of the polymer chains due to high temperature, possible CNT–p-aramid interactions. Absorptions at 862, 821, 655, and 521 cm^−1^ are associated with out-of-plane C–H bending in aromatic rings. These bands appear slightly altered (either shifted or with minor intensity differences) in the gCPO spectrum, which may indicate microstructural perturbations in the aramid backbone CNT deposition residues. Based on these findings, it was concluded that the peaks associated particularly with aromatic C=C stretching, C–H bending, and characteristic CNT vibrational modes were altered following CNT growth via the CVD process. Moreover, this interpretation is supported by the pull-out test results of the CNT-grown aramid specimens. The lower tensile performance of the CNT-grown yarns compared to the control counterparts is also attributed to the structural alterations induced by the CVD treatment.

#### 3.1.3. Raman Spectroscopy Results

**Figure 8:**
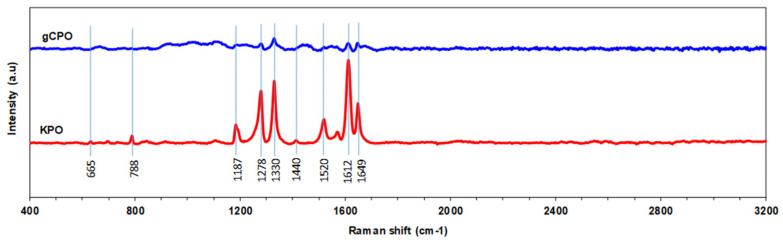
Raman spectroscopy data of CNT-grown p-aramid substrate and neat p-aramid fabric.

Raman spectra of pristine para-aramid fabric (KPO) and CNT-grown para-aramid fabric (gCPO) are presented in Figure 8. Both samples display multiple peaks between 500 and 3000 cm^−1^, corresponding to characteristic molecular vibrations of aromatic polyamides and carbonaceous species. In the pristine p-aramid spectrum, distinct bands are observed at 788, 1187, 1278, 1329, 1413, 1519, 1612, and 1648 cm^−1^, which correspond, respectively, to C–H out-of-plane bending, C–C ring stretching, C–N stretching, and amide-related vibrations of the aromatic backbone. These results are consistent with literature reports for Twaron-type para-aramid structures [70,71].

As illustrated in Figure 8, the Raman peak at 665 cm^−1^ is attributed to C–C bending vibrations within the p-aramid molecular backbone. The 788 cm^−1^ peak is associated with out-of-plane C–H bending vibrations in the aromatic ring structures. CNT growth may induce pronounced variations in the intensity of this peak due to structural and interfacial alterations. The 1187 cm^−1^ Raman band corresponds to in-plane bending vibrations of C–H groups within the aromatic rings of para-aramid filaments [70,71], and may reflect surface-level interactions following CNT deposition. The 1278 cm^−1^ peak is assigned to the C–N stretching vibrations of amide groups. CNT growth is likely to influence these vibrations through interactions with the amide linkages, potentially causing peak shifts or changes in intensity [70,71]. The 1520 cm^−1^ peak corresponds to C=C stretching vibrations in the aromatic rings of the p-aramid. This peak’s intensity and position are modulated by the presence of CNTs, indicating structural perturbations introduced during CVD treatment [72]. Furthermore, the gCPO sample reveals two additional dominant peaks at approximately 1346 cm^−1^ (D band) and 1585 cm^−1^ (G band), characteristic of carbon nanotubes [73]. The D band may arise from sp^3^ carbons mode or lattice defects, while the G band corresponds to the in-plane vibrations of sp^2^-bonded carbon atoms in the structure, confirming the formation of multi-walled CNTs (MWCNTs) [73]. According to Kharlamova et al. [73], ID/IG intensity ratio (1.12) are typical for MWCNTs synthesized at 400–500 °C, where simultaneous nucleation and carbon formation occur. A slight shift in the G band from 1612 cm^−1^ in KPO to 1585 cm^−1^ in gCPO may be attributed to thermal relaxation between the CNT wall and the aramid fiber. Additionally, the 1649 cm^−1^ Raman peak corresponds to the C=O stretching vibrations of amide functionalities [71]. The coexistence of CNT-related and aramid-related peaks implies that CNTs are physically anchored onto the fiber surface without complete carbonization of the underlying polymer. On the other hand, based on the mass-difference analysis summarized in Table 2, the residual Fe/Co catalyst fraction remaining on the para-aramid substrate after the CVD process was estimated to be below 0.1 wt.%. No additional Raman bands associated with Fe–O or Co–O vibrations (typically appearing near 560–680 cm^−1^) were detected, indicating that the residual catalyst amount was below the spectroscopic detection threshold. Furthermore, the FTIR spectra maintained the characteristic amide (C=O, C–N) and aromatic (C=C) vibration peaks of the para-aramid structure, confirming that the trace catalyst residues did not chemically interact with or alter the polymer backbone. Therefore, while minor Fe/Co particles may remain embedded within the CNT network—as commonly observed in low-temperature CVD processes—their influence on the overall spectral features and chemical integrity of the para-aramid fabric is considered negligible.

Overall, Raman spectroscopy confirms successful CNT deposition and the formation of a hybrid sp^2^–sp^3^ carbon network on the aramid surface. The observations align with literature, indicating that Raman signatures of MWCNTs are governed by the degree of crystallinity, defect density, and substrate–CNT interfacial interactions.

#### 3.1.4. X-Ray Diffraction (XRD) Results

**Figure 9:**
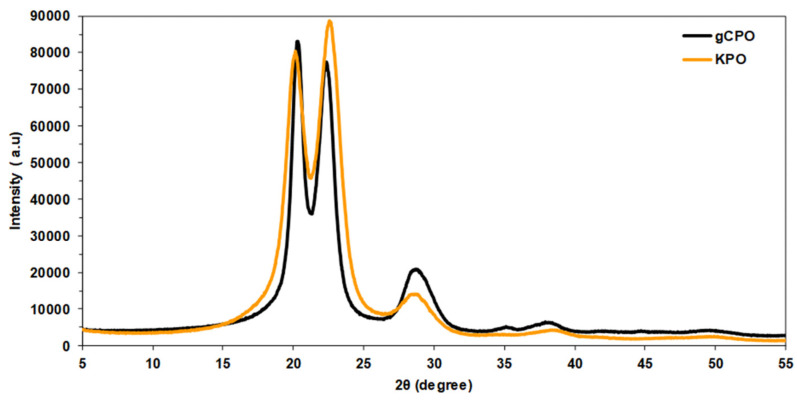
X-Ray diffraction (XRD) spectra of CNT-grown and pristine para-aramid fibers.

XRD patterns of pristine (KPO) and CNT-grown (gCPO) para-aramid specimens are presented in Figure 9. The main reflections observed for the control KPO sample occur at 2θ as 20.137°, 22.552° and 28.881° (intensities: 80337, 88590 and 13988, respectively), while the gCPO sample shows peaks at 2θ as 20.294°, 22.316° and 28.697° (intensities: 83074, 77504 and 20970, respectively). These peak positions and their systematic intensity/shape changes upon CVD treatment indicate coexisting contributions from the para-aramid crystalline domains and newly formed carbonaceous phases after CNT growth, as discussed by Yalovega et al. [74].

In peaks at ~20.1–20.3° and ~22.3–22.6° (KPO/gCPO), these reflections are assigned primarily to the semi-crystalline ordering of the para-aramid backbone. The small shift in 2θ (≈0.15–0.25°) and the relative intensity decrease at 22.3° in gCPO compared to KPO are consistent with partial disruption of long-range polymer order caused by the high-temperature CVD treatment and by the presence of surface-grown CNTs. The FTIR and Raman data corroborate partial modification of the polymer backbone and an increase in surface carbonaceous disorder, supporting this interpretation [74]. In peak near ~28.7–28.9°, the enhanced intensity and relative sharpening of the 28.7° reflection in gCPO (compared to 28.88° in KPO) likely arise from contributions of deposited CNTs (mainly, short and thick MWCNTs) that overlap with polymer features. Therefore, we attribute the increased intensity at ~28.7° in gCPO largely to CNTs contributions, while acknowledging a possible small overlap with polymer-related scattering [74]. This explanation is consistent with the Raman and the FESEM observation of short/wide CNTs on filaments. The minor 2θ shift (from 20.137° to 20.294°) can be attributed to a slight reduction in lattice spacing, reflecting thermally induced contraction within the semi-crystalline para-aramid domains during the 450 °C CVD process. Simultaneously, the enhanced reflection near ≈28.7° is consistent with the multi-walled CNTs, indicating partial overlap between the polymeric crystalline peaks and graphitic contributions. Therefore, the observed peak modifications arise from a combined effect of localized thermal relaxation and CNT deposition rather than from complete structural degradation. In conclusion, the CNT growth process distinctly modified the structural characteristics of the gCPO samples. This modification led to enhanced pull-out performance compared to the control specimens. However, a notable reduction in tensile strength was observed.

#### 3.1.5. Thermogravimetric Analysis (TGA/DTA) Results

**Figure 10:**
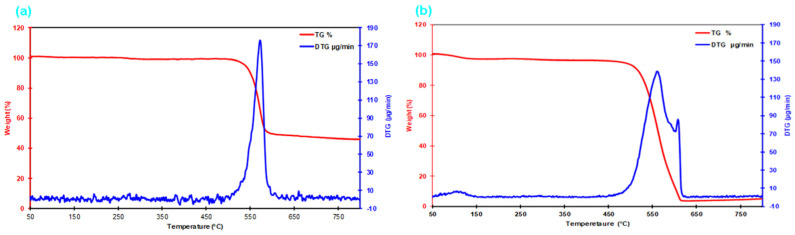
Thermogravimetric analysis (TGA) results of the CVD-processed para-aramid fiber surface with CNT growth. (**a**) CNT-grown para-aramid fiber and (**b**) untreated para-aramid fiber.

**Figure 11:**
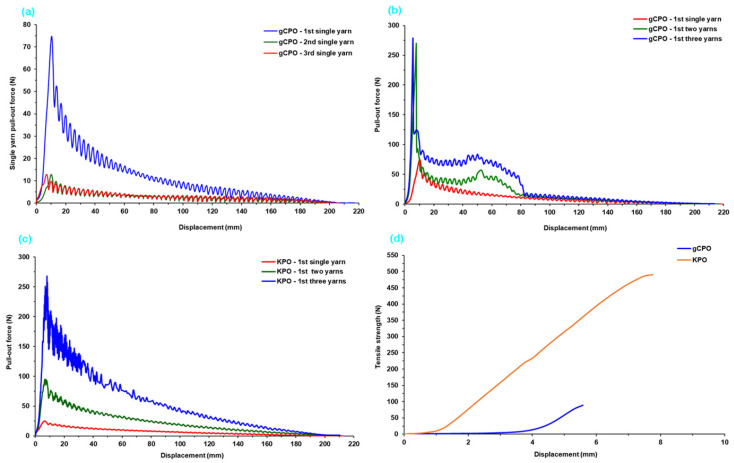
Force (N)–displacement (mm) profiles corresponding to single- and multiple-yarn pull-out tests for CVD-treated p-aramid substrates. (**a**) Successive single-yarn pull-out behavior of CNT-grown p-aramid fabric (gCPO), (**b**) multiple-yarn pull-out response of CNT-grown fabric, (**c**) multiple-yarn pull-out performance of neat fabric (KPO), and (**d**) comparative tensile strength characteristics of CNT-functionalized and neat p-aramid yarns.

The thermogravimetric (TGA/DTA) profiles of CNT-grown and neat p-aramid fabric structures via chemical vapor deposition (CVD) are presented in Figure 10a,b. When evaluating the TG/DTG profiles of the CNT-grown para-aramid filaments in comparison with the neat filaments, it is evident from Figure 10a,b that the para-aramid fiber exhibited a 1% weight loss at the CNT growth temperature of 450 °C. Furthermore, at 554 °C and 576 °C, the para-aramid fiber showed additional weight losses of 13% and 40%, respectively. At 600 °C, the CNT-grown structure retained approximately 49.59% of its initial mass. Additionally, the onset decomposition temperature for CNT-grown para-aramid fibers was recorded at 556 °C, indicating the initial release of volatile components corresponding to a 15% mass loss. The maximum rate of thermal degradation, as identified by the peak of the derivative thermogravimetric (DTG) curve, occurred at 574 °C, representing the temperature at which the decomposition rate reached its maximum. On the other hand, while the CNT-grown structure demonstrated enhanced pull-out performance (Figure 11a,b), the single-yarn tensile strength was significantly lower than that of the control sample, which is likely attributed to the high-temperature effects of the CVD process (Figure 11d).

### 3.2. Pull-Out Properties

#### 3.2.1. Single- and Multiple-Yarn Pull-Out Force–Displacement Results

The force (N)–displacement (mm) data obtained from single- and multiple-yarn pull-out tests of CNT-grown and control p-aramid fabric are presented in Table 3. Additionally, Figure 11a–d illustrates the successive single- and multi-yarn (2-end and 3-end) pull-out force–displacement profiles for the CNT-grown p-aramid fabric structures. These experimental results were generated to investigate the influence of CNT growth on the filament surfaces, particularly focusing on the alterations in friction-driven single- and multiple-yarn pull-out forces at the fabric interface. The aim is to elucidate the relationship between these interfacial mechanical interactions and the overall energy dissipation behavior of the CNT-grown on the fabric surface.

The single-yarn pull-out curve typically exhibits an initially mild non-linear increase in force, culminating near the peak pull-out load-an interval commonly referred to as the crimp extension phase (stage I). This region corresponds to the elongation of yarn segments engaged in warp–weft interlacements, which are fundamental to the fabric formation, and includes the initial onset of frictional resistance. Following this, the second phase—termed the initial interlacement rupture zone—is characterized by a noticeable force drop immediately after the maximum pull-out load, indicating the initial disengagement of the warp from the transverse (filling) yarn (stage II). The final phase, defined as the stick–slip or translation region, displays a fluctuating force response with alternating decreases and increases, ultimately transitioning to a quasi-steady-state as the warp progressively disengages from successive filling (stage III) [75].

Based on the pull-out force data presented in Table 3 and Figure 11a–d, it was determined that the single-, double-, and triple-yarn pull-out forces of the CNT-grown para-aramid structure (gCPO) were approximately 66.19%, 2.84-fold, and 3.81% higher, respectively, compared to the control para-aramid fabric (KPO). The in situ growth of carbon nanotubes (CNTs) on the para-aramid filaments likely led to more effective anchoring of the filaments within the warp and weft yarn bundles, resulting in the formation of roughened or CNT-fibered filament surfaces [50]. This modified surface topology appears to enhance frictional interactions at the yarn–yarn interface, particularly among CNT-decorated filaments. Consequently, the CNT-functionalized filaments demonstrated increased resistance to axial extraction, especially prior to the onset of the initial interlacement rupture zone, thereby yielding higher maximum pull-out forces. Compared with the neat para-aramid control (KPO), the CNT-grown yarns displayed a distinctly modified stick–slip behavior. As seen in Figure 11b,c, the gCPO samples exhibited more frequent stick–slip oscillations with smaller force-drop amplitudes, implying a smoother yet more energy-intensive sliding process. This shift can be attributed to the CNT-induced nanoroughness and increased inter-filament adhesion, which generate numerous micro-pinning points along the contact interface. These micro-asperity junctions intermittently form and rupture during yarn extraction, yielding a higher stick–slip frequency and a broader energy dissipation window. The cumulative effect of these events enhances inter-yarn frictional stability and correlates with the higher stick–slip energy especially in single-yarn pull-out (Table 4). Conversely, the tensile strength of single yarns extracted from the control KPO structure exhibited a substantial increase of approximately 563% in comparison to those from the gCPO structure. As shown in Figure 11d, the CNT-grown yarns displayed markedly lower tensile performance. This typical degradation in tensile strength is attributed to thermal damage at the molecular level within the para-aramid filaments during high-temperature CNT growth via CVD. Therefore, it is advisable to conduct processing at lower temperatures for para-aramid filament-based soft fabric systems and to investigate alternative low-temperature nanostructure deposition techniques such as cold-growth nanofiber synthesis to preserve the intrinsic mechanical properties.

#### 3.2.2. Initial Interlacement Rupture Results

**Figure 12:**
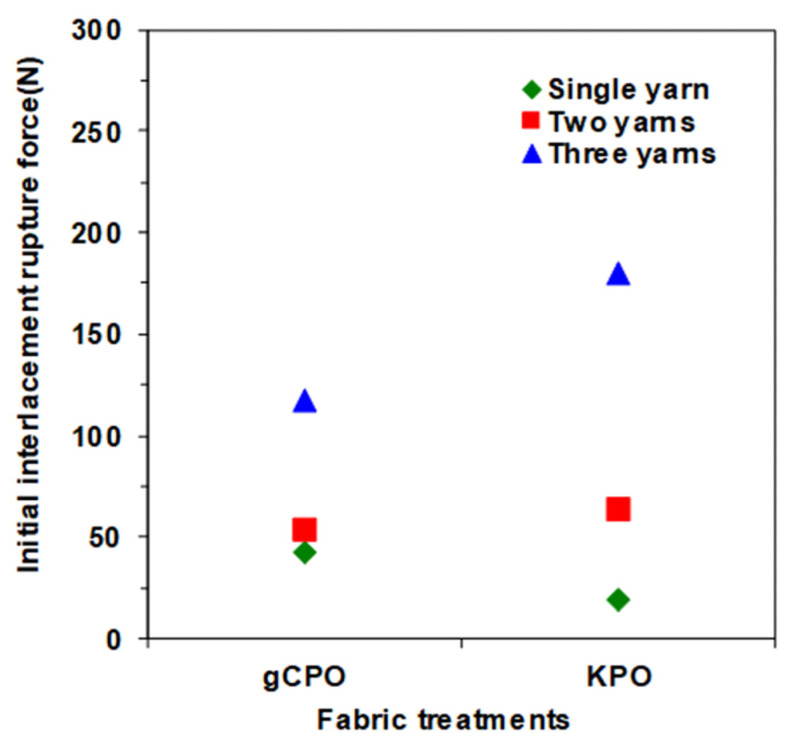
Initial interlacement rupture forces for CNT-grown p-aramid substrate.

Figure 12 illustrates the initial interlacement rupture forces recorded for both single- and multiple-yarn pull-out configurations in CNT-functionalized p-aramid fabric systems. The initial interlacement rupture force is characterized by a sharp force drop immediately following the peak pull-out load, corresponding to the disengagement of the warp tow from its mechanical interaction with the transverse (weft) tow. For analytical clarity, the transient stick–slip phase occurring after this event was excluded from the present evaluation.

As illustrated in Figure 12, the initial interlacement rupture forces for both CNT-grown (gCPO) and control (KPO) fabric systems ranged from 19.20 to 145.28 N. The single-yarn pull-out configuration of the gCPO specimens exhibited a pronounced enhancement of 55.32% in rupture force relative to the KPO counterparts, reflecting improved interfacial adhesion. In the case of multi-yarn pull-out, the gCPO fabric demonstrated enhanced mechanical performance, with the two-yarn configuration achieving an 18.20% increase in rupture resistance over KPO. However, a reduction of 23.91% was observed in the three-yarn configuration compared to the control group. These outcomes underscore the functional influence of CNT incorporation on the interfacial mechanics of aramid substrate and further highlight the imperative for process-level optimization strategies that align with application-specific mechanical performance requirements.

The CNT-grown para-aramid fabric exhibited enhanced single- and two-yarn interlacement rupture forces, primarily due to the synergistic interactions between the carbon nanotubes and the filament surfaces, as well as their cumulative effect within the interlacement zones of the fabric structure. These interactions increased frictional resistance at the yarn–yarn interfaces, thereby contributing to improved pull-out force during the initial rupture phase. In contrast, the interlacement rupture forces observed during three yarn pull-out tests was lower in the CNT-functionalized fabric compared to the KPO structure. This reduction is predominantly attributed to the thermal degradation of the molecular structure of p-aramid fibers during the high-temperature CVD process-a finding also evidenced by the significant decrease in tensile strength of CNT-grown yarns. Consequently, when the increase in friction-based yarn–yarn resistance during multi-yarn pull-out surpasses the compromised tensile strength threshold of the yarns, premature filament rupture is triggered. This mechanical failure negatively influences the overall pull-out performance in multi-yarn configurations. Therefore, the selection of CVD process parameters becomes critical. It is imperative to conduct CNT growth at optimized temperatures that do not compromise the internal molecular architecture of p-aramid substrates. Achieving CNT deposition without inducing thermal damage to the reinforcement medium should be a central focus in future research. In addition, several structural and geometrical parameters significantly influence yarn interlacement rupture forces, including the interlacement pattern, the number and type of interlacements [76], the directional linear densities of the yarns [50], fabric thickness and dimensions [77], and overall fabric density [78].

#### 3.2.3. Intra-Yarn Shear Strength Results

**Figure 13:**
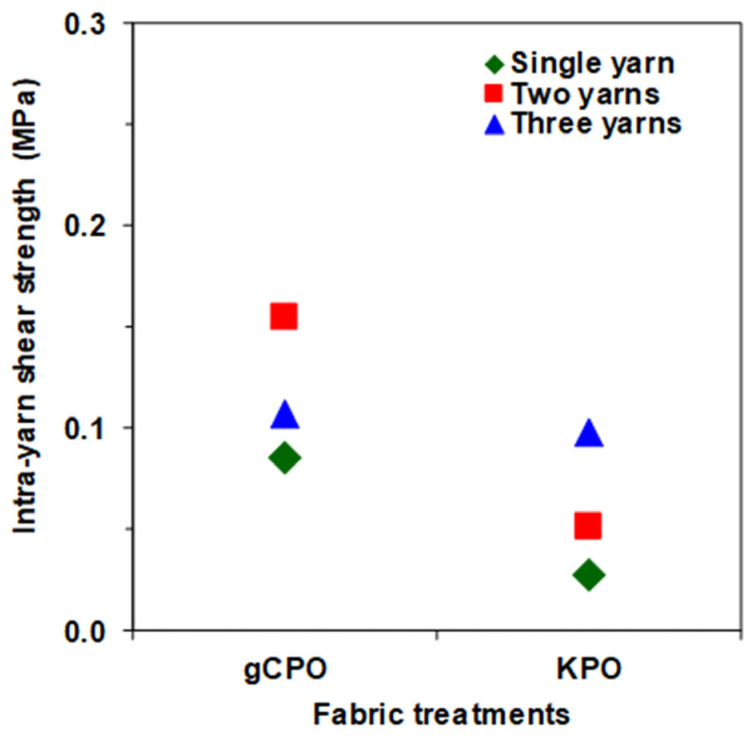
Intra-yarn shear strengths via pull-out for CNT-grown p-aramid substrate.

The intra-yarn shear strength values for both single- and multiple-yarn pull-out configurations of the gCPO fabric are presented in Table 3, while Figure 13 graphically illustrates the corresponding force responses for the gCPO sample. As evidenced in Table 3 and Figure 13, the intra-yarn shear strength for both the gCPO and its unmodified sample (KPO) ranges between 0.03 MPa and 0.16 MPa across single-, two-, and three-yarn pull-outs. Notably, the gCPO fabric demonstrated a substantial enhancement in single-yarn shear strength, exhibiting a three-fold increase relative to the KPO structure. Furthermore, in the two- and three-yarn configurations, the intra-yarn shear strength of the gCPO surpassed that of the KPO by approximately 3.2-fold and 9.1%, respectively.

These mechanical improvements can be primarily ascribed to the incorporation of grown carbon nanotubes within the aramid substrate surface, which effectively augment frictional interactions at the yarn interlacement zones and filament contact surfaces during the crimp-extension phase. This structural modification enhances the shear load transfer capability by increasing resistance to inter-yarn slippage [34].

Critical factors influencing intra-yarn shear strength include, but are not limited to, fabric areal density in the principal directions [78], interlacement topology [78], geometric parameters such as fabric thickness and unit cell dimensions [77], and the linear mass density of constituent yarns in warp and weft directions [79]. Importantly, consistent with the scope of this investigation, the impact of CNT growth and yarn terminal conditions on localized intra-yarn shear strength, particularly regarding warp-oriented pull-out resistance and angular interfacial strength at warp-weft intersections was thoroughly examined through quantitative analysis and comparative evaluation of force–displacement responses.

#### 3.2.4. Pull-Out Energy Results

**Figure 14:**
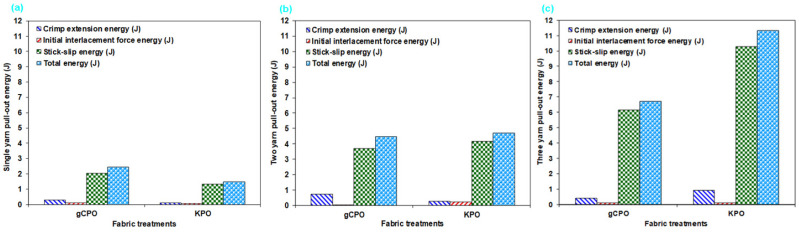
Pull-out energy of CNT-grown p-aramid fabric substrates. (**a**) Single-yarn pull-out energy, (**b**) two yarn pull-out energy and (**c**) three yarn pull-out energy.

The single- and multiple-yarn pull-out energy data of CNT-grown p-aramid fabric are tabulated in Table 4. Furthermore, Figure 14 illustrates the energy dissipation components observed during pull-out tests of the gCPO fabric surface including crimp extension energy (J), initial interlacement rupture force energy (J), and stick–slip frictional energy [75].

In Table 4, the contribution of distinct energy components to the total energy absorption during single-yarn pull-out tests—namely, crimp extension energy, initial interlacement rupture force energy, and stick–slip energy was quantitatively assessed. For the KPO specimen, these contributions were calculated as 6.67%, 4.00%, and 89.33%, respectively. In contrast, the gCPO configuration exhibited corresponding contributions of 12.30%, 4.91%, and 83.20%. In the context of dual-yarn pull-out experiments conducted on all processed soft fabric configurations, the crimp extension energy contribution to the overall energy dissipation ranged from 5.97% to 16.52%, whereas the contribution of the initial interlacement rupture force energy varied between 1.12% and 4.90%. Notably, stick–slip energy emerged as the dominant dissipation mechanism, with its contribution fluctuating between 82.59% and 89.34%. Moreover, in triple-yarn pull-out evaluations, the crimp extension energy accounted for 6.27% to 8.04% of the total absorbed energy, while the initial interlacement rupture force energy contribution was recorded in the range of 0.97% to 1.64%. The stick–slip energy consistently remained the principal component, contributing between 91% and 92.1%.

As depicted in Figure 14a–c, the total single-yarn pull-out energy of the gCPO structure was quantified to be 38.53% higher than that of the KPO counterpart. However, in the dual- and triple-yarn pull-out, the total pull-out energies for gCPO were determined to be 4.69% and 68.96% lower, respectively, compared to the KPO structure. Although gCPO exhibited superior energy dissipation performance under single-yarn pull-out conditions, its performance significantly deteriorated under multi-yarn pull-out loading. This behavior is presumably attributable to the adverse effects of elevated processing temperatures during CNT growth on molecular-level chemical interactions. These interactions likely compromised the tensile strength of the filament bundles (Figure 11d). Furthermore, the presence of a larger number of yarn terminals in the gCPO configuration intensified inter-fiber friction during multi-yarn pull-out, thereby promoting premature filament fracture.

In summary, among the distinct energy dissipation zones identified in the CNT-functionalized fabric systems, the dominant contributions to total pull-out energy listed in descending order were attributed to stick–slip energy, crimp extension energy, and initial interlacement rupture force energy. It can be inferred that CNT-grown para-aramid structures exhibit enhanced energy absorption in single-yarn pull-out modes across all dissipation zones. However, their performance is markedly diminished in multi-yarn pull-out scenarios when compared to the untreated control samples.

#### 3.2.5. Pull-Out Fracture Toughness Results

**Figure 15:**
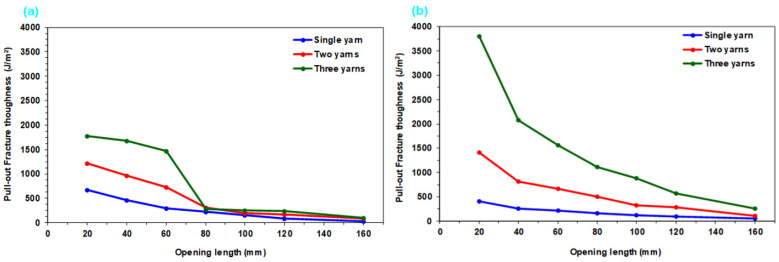
Single- and multiple-yarn pull-out fracture toughness-fabric opening length of CVD-processed p-aramid fabric substrate. (**a**) CNT-grown p-aramid substrate (gCPO) and (**b**) neat fabric substrate (KPO).

**Figure 16:**
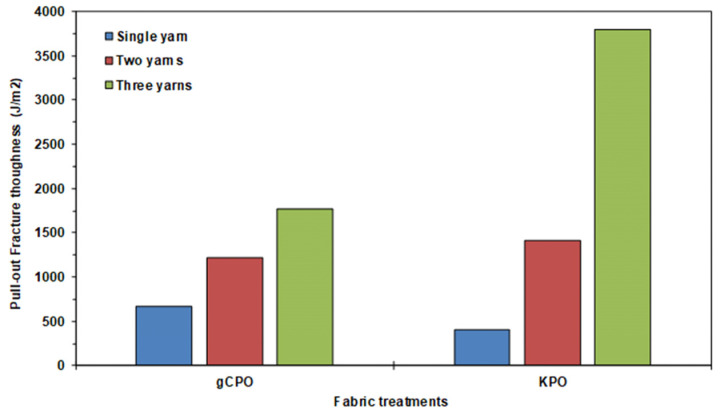
Maximum pull-out fracture toughness of CNT-grown and neat p-aramid substrates.

Table 5 presents computed single- and multiple-yarn pull-out fracture toughness of CNT-grown and neat p-aramid substrate considering Equation (7). The toughness of substrates was assessed by analyzing single- and multiple-yarn pull-out fracture toughness during the pull-out load in the stick–slip phase. Fabric openings at 20 mm intervals were considered, with relevant parameters—stick–slip pull-out, displacement, fabric width, and opening length—quantified using Equation (7). The findings demonstrate that Equation (7) is a reliable method for characterizing the fracture toughness of CNT-grown substrate. Accumulated retraction forces from warp disintegration [80,81], and minor yarn slippage or fabric shear [34] were excluded from this analysis. Additionally, Figure 15 illustrates single- and multiple-yarn pull-out fracture toughness (J/m^2^)-fabric opening length (mm) of CNT-grown and neat substrates. Figure 16 presents a comparative assessment of single- and multi-end pull-out fracture toughness across CNT-grown and neat p-aramid substrates.

As shown in Table 5 and Figure 15a,b, the pull-out fracture toughness of CNT-grown and neat substrates ranges from 408.74 to 670.70 J/m^2^ for single-end yarns and from 1769.29 to 3797.21 J/m^2^ for three-end yarns. Generally, fracture toughness decreases slightly with increasing opening length, while higher toughness values were observed with an increased number of yarn ends across all substrates. This trend is attributed to disintegration at the warp-filling interlacement points, where friction, interlacement patterns, and fabric density-induced lateral pressure play crucial roles. Similar non-linear increases in pull-out forces with more yarn ends have been reported in previous studies [60]. Furthermore, predictive techniques such as finite element modeling (FEM) and deep learning, and machine learning-based artificial neural networks (ANN) offer strong potential for future investigations into these behaviors.

As depicted in Figure 16, the peak fracture toughness attained in the single-yarn pull-out in gCPO exhibited a 39.06% enhancement relative to the KPO. Conversely, for the two- and three-yarn pull-out scenarios, the gCPO samples demonstrated reductions in fracture toughness by 16.26% and 214.62%, respectively, when compared to the KPO counterparts. These findings indicate that the incorporation of CNTs grown via CVD improves the interfacial energy dissipation during single-yarn extraction but adversely impacts the fracture toughness under multiple-yarn pull-out conditions.

This discrepancy is likely attributed to the elevated interfacial frictional resistance that arises between the CNT-coated filaments and among the CNTs themselves as the number of pull-out yarns increases. This heightened resistance can exceed the intrinsic tensile strength of the filaments, thereby inducing premature or multiple filament fractures during the extraction process. Furthermore, as observable in Figure 11d, the CVD process may have led to molecular-level degradation within the filament structure, further diminishing their inherent tensile properties. Notably, the fracture toughness of fabric structures under pull-out loading is governed by a multitude of structural parameters, including the interlacement architecture [9,76,78], directional yarn density [9,79,82], and the yarn linear density [9,77].

### 3.3. Nano Friction Properties

#### 3.3.1. Static Friction Results

**Figure 17:**
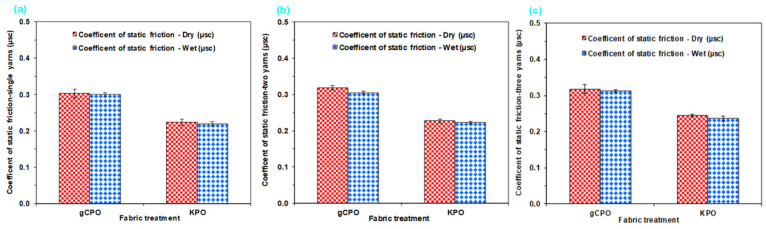
Static friction coefficients of CVD-treated p-aramid fabric structures under dry and wet conditions for single- and multiple-yarn configurations. (**a**) Static friction coefficient of single yarn, (**b**) static friction coefficient of dual-yarn system, and (**c**) static friction coefficient of triple-yarn system.

The static coefficients of friction were obtained from surface-grown carbon nanotube-functionalized p-aramid structures under both dry and wet conditions via chemical vapor deposition (CVD). Raw frictional force data were acquired using a customized device based on the capstan principle. The corresponding friction coefficients were subsequently calculated using the classical Euler’s Equation (8). In parallel, kinetic friction coefficients in the dry state were extracted from the adhesion–sliding transition regions of pull-out force (N) versus displacement (mm) curves. The static and kinetic friction coefficients derived from the CNT-grown fabric specimens under both environmental conditions are summarized in Table 6. Furthermore, Figure 17a–c illustrates the static frictional behavior of single- and multiple-yarn systems coated with CNTs via CVD, presenting comparative results for dry and wet configurations.

As illustrated in Figure 17a–c, the static friction coefficients for all surface-modified fabric structures in the single-yarn configuration were observed to range between 0.220 and 0.300 under both dry and wet conditions. In the dual-yarn configuration, the static friction coefficients varied between 0.230 and 0.320 in the dry state, and between 0.223 and 0.305 under wet conditions. Furthermore, in the triple-yarn arrangement, static friction coefficients were recorded within the range of 0.250 to 0.320 for dry environments and 0.240 to 0.310 for wet environments.

The dry static friction coefficients of the single-, double-, and triple-yarn configurations in the gCPO structures were found to be higher by 26.67%, 28.13%, and 21.88%, respectively, compared to those of the KPO counterparts. The highest dry friction coefficients were observed in both single- and multiple-yarn systems incorporating CNT-grown architectures. This enhancement is attributed to the increase in contact and interaction areas resulting from the surface roughening effect caused by the protruding carbon nanotubes on the filament surfaces (Figure 6c,d). Consequently, the elevated dry friction performance is ascribed to the enhanced interfacial interactions between nanotube-nanotube and nanotube-filament interfaces.

The wet static friction coefficients of the single-, double-, and triple-yarn configurations in the gCPO structures were found to be higher by 26.67%, 26.89%, and 22.58%, respectively, compared to those of the KPO structures. The highest wet friction coefficients were observed in both single- and multiple-yarn configurations with CNT-grown yarns. This phenomenon is attributed to the increase in interfacial contact areas caused by the nano-scale protrusions of carbon nanotubes, which induce surface fibrillation on the filaments (Figure 6c,d). As a result, enhanced frictional resistance is primarily governed by intensified nanotube-nanotube and nanotube-filament surface interactions under wet conditions. While the single-yarn systems displayed nearly identical μsc values, a slight reduction (Δμ ≈ 0.01–0.015) was detected for the dual- and triple-yarn configurations, suggesting partial surface wetting and minor capillary effects at localized contact junctions. Consequently, the comparable dry/wet friction response implies that the CNT network sustains stable interfacial friction even in humid or aqueous environments, enhancing the environmental robustness of the nano-functionalized fabrics. Moreover, the dry and wet friction coefficients of the modified soft fabric systems exhibited similar variation trends. Additionally, the number of yarn ends was found to have no significant or anomalous influence on the static friction coefficients under either dry or wet testing environments.

#### 3.3.2. Kinetic Friction Results

**Figure 18:**
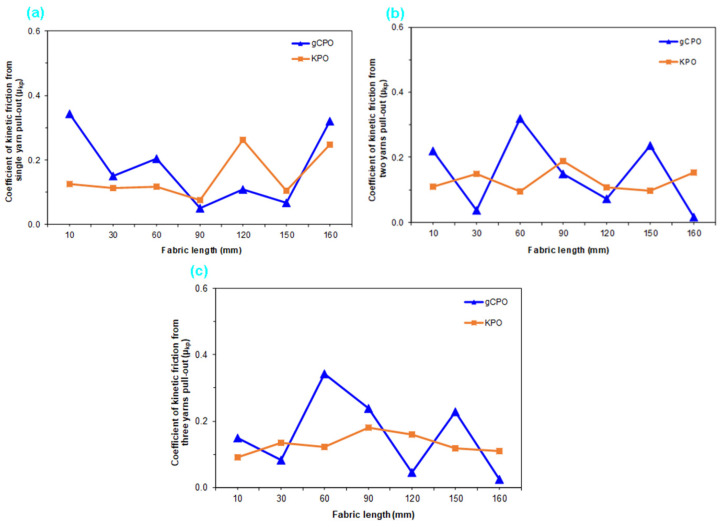
Dry kinetic friction coefficients of CVD-treated p-aramid structures for single- and multiple-yarn configurations. (**a**) Kinetic friction coefficient of single-yarn system, (**b**) kinetic friction coefficient of dual-yarn system, and (**c**) kinetic friction coefficient of triple-yarn system.

Figure 18 presents the dry kinetic friction coefficients for single- and multiple-yarn configurations in CVD-treated and control p-aramid fabric structures. As illustrated in Figure 18a–c, the dry kinetic (dynamic) friction coefficients of single-yarn configurations in CNT-grown and control fabric structures ranged between 0.150 and 0.180. In contrast, the dual-yarn systems exhibited dry kinetic friction coefficients in the range of 0.130 to 0.150. Furthermore, for the triple-yarn configurations, the dynamic friction coefficients under dry conditions were determined to vary between 0.130 and 0.160.

The dry dynamic friction coefficient (μ_kp_) of the single-yarn configuration in the gCPO structures was found to be 16.67% higher compared to that of the KPO. Similarly, the dual- and triple-yarn configurations of the gCPO structures exhibited increases in dry dynamic friction coefficients ranging from 13.33% to 18.75% relative to the KPO structures. The highest dry dynamic friction coefficients were recorded in both single- and multiple-yarn systems incorporating CNT-grown architectures. The elevated dynamic friction coefficients in single- and multi-yarn configurations are attributed to the CNTs grown on the p-aramid filament surfaces, which increase the effective contact area and surface roughness compared to the control fabrics. Furthermore, a comparative assessment between the dry static and kinetic friction coefficients of the single-yarn configuration revealed that the kinetic friction coefficient was consistently lower than its static counterpart. This trend, while indirectly influenced by the filament surface modification, is fundamentally associated with the continuous sliding-based frictional mechanism under high contact loads in dynamic friction events, as also reported in the open literature.

### 3.4. Nanocoated Fabric Pull-Out Mechanism

**Figure 19:**
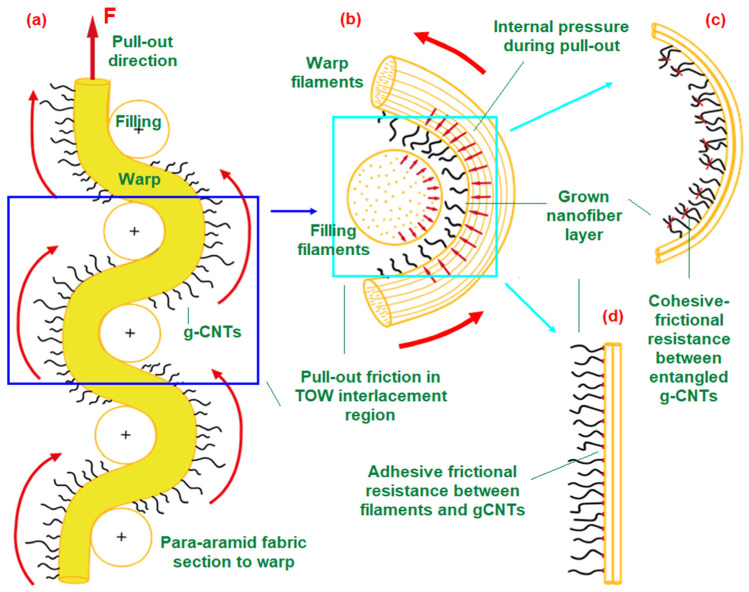
Schematic illustration of the gCNTs fabric pull-out mechanism. (**a**) gCNTs para-aramid fabric at the initial stage of pull-out, (**b**) displacement of gCNTs within the warp–filling interlacement region under pull-out loading, (**c**) interfacial pull-out frictional resistance between entangled gCNTs, and (**d**) pull-out frictional resistance at filament/gCNTs interfaces.

Figure 19a–d schematically illustrates the pull-out mechanism of carbon nanotube-grown (gCNT) fabrics. As observed, when a pull-out force is applied to the warp direction of the gCNT fabric at its initial state (Figure 19a), the gCNT segment of the filament tow is displaced toward the warp–filling interlacement region (stage I). This displacement, combined with the local warp–filling contact pressures, enhances friction-based resistance between adjacent filament tows (Figure 19b). In gCNTs, such resistance is presumed to arise from contact-based cohesive interactions, attributed to multiple nanotube–nanotube entanglements (Figure 19c), together with adhesive interactions at the filament/gCNT interfaces (Figure 19d). Moreover, due to compressive forces generated in the warp–filling interlacement region during pull-out, the transferred gCNTs are expected to undergo flexural along the filament length and buckling-induced deformations depending on the grown tube length. These deformation modes further augment the frictional resistance between gCNTs and filaments. Consistent with the pull-out results (Figure 11, Table 3), gCNTs exhibited significantly higher pull-out resistance compared to the control.

## 4. Conclusions

Carbon nanotubes (CNTs) were synthesized on para-aramid fabrics (gCPO) via the chemical vapor deposition (CVD) process to evaluate their influence on pull-out behavior, fracture toughness, and frictional performance. The CNTs exhibited a curved morphology with relatively short lengths and thick tubular diameters on the filamentary substrates. Spectroscopic analyses (FTIR, Raman) and partial shifts in XRD peaks confirmed that CNT growth altered the molecular structure of the para-aramid filaments. Thermogravimetric analysis (TGA) further indicated that these structural modifications were influenced by the elevated thermal loads associated with CVD processing.

CNT-functionalized filaments demonstrated increased axial extraction resistance, particularly in the pre-interlacement rupture stage, yielding higher maximum pull-out and interlacement rupture forces compared to untreated control (KPO) fabrics. Conversely, single yarns from the control structure exhibited higher tensile strength than those from gCPO, suggesting partial thermal degradation during CNT synthesis. The enhanced intra-yarn shear strength in gCPO is attributed to CNT integration on filament surfaces, which increases contact area and frictional engagement within yarn interlacement zones during the crimp-extension phase, thereby improving shear load transfer and resisting inter-yarn slippage.

CNT-grown para-aramid structures exhibited superior energy absorption and fracture toughness in single-yarn pull-out tests; however, performance decreased under multi-yarn pull-out due to excessive frictional resistance surpassing the intrinsic tensile strength of the filaments. Static and kinetic friction coefficients increased in gCPO fabrics, consistent with surface roughening from protruding CNTs, which enlarged contact and interaction areas. For future optimization, lower-temperature processing or alternative nanostructure deposition methods—such as cold-growth nanofiber techniques—are recommended to preserve the intrinsic mechanical integrity of para-aramid filaments while retaining the advantages of CNT functionalization.

## Figures and Tables

**Table 1:** Carbon nanotube (CNT)-grown para-aramid fabric surface tailored for yarn pull- out performance evaluation.

Sample Code	Structures	Number of Layer	Nano Material and Ratio (%)
KPO	Control fabric	1	-
gCPO	CNT-grown p-aramid fabric	1	CNTs (~0.315)

**Table 2:** Mass ratios characterizing carbon nanotubes grown in situ on para-aramid substrate surfaces.

Sample Code	Fabric Weight (W_d_, g)	Weight of the Fabric After Chemical Removal (W_df_, g)	Catalyst-Treated Fabric Weight (W_cf_, g)	Weight of the Fabric and Catalyst with Grown Carbon Nanotubes (W_gf_, g)	Fabric Weight After Thermal Treatment (W_loss_,450 °C, g)	Catalyst Mass Deposited on the Fabric (W_ct_, g)	Effective Mass of the Carbon Nanotube-Integrated Fabric (W_gfeff_, g)	Weight Percentage of Carbon Nanotubes (W_vf_, %)
gCPO	18.506	18.118	18.215	17.964	17.811	0.097	17.867	0.315

**Table 3:** Single- and multiple-yarn peak pull-out force–displacement data, initial interlacement rupture force, and intra-yarn shear strength values for CNT-grown para-aramid substrate.

Label	Pull-Out Force (Max, N)	Displacement (mm)	Initial Interlacement Rupture Force (N)	Intra-Yarn Shear Strength (MPa)	Yarn Tensile Strength (N, N/tex)
Single yarn					
gCPO	74.66	10.28	42.97	0.09	87.63 ± 9.18
KPO	25.24	6.49	19.20	0.03	493.10 ± 19.78
Multiple yarns					
Two yarns					
gCPO	269.22	7.91	77.47	0.16	-
KPO	94.79	7.11	63.37	0.05	-
Three yarns					
gCPO	278.48	5.59	117.75	0.11	-
KPO	267.88	8.14	145.28	0.10	-

**Table 4:** Pull-out energy of CNT-grown and control substrate results.

Label	Crimp Extension Energy (J)	Initial Interlacement Rupture Force Energy (J)	Stick–Slip Energy (J)	Total Energy (J)
Single yarn				
gCPO	0.30	0.12	2.03	2.44
KPO	0.10	0.06	1.34	1.50
Multiple yarns				
Two yarns				
gCPO	0.74	0.05	3.70	4.48
KPO	0.28	0.23	4.19	4.69
Three yarns				
gCPO	0.42	0.11	6.17	6.70
KPO	0.91	0.11	10.30	11.32

**Table 5:** Single- and multiple-yarn pull-out fracture toughness of CNT-grown p-aramid substrate.

Label	Fabric Opening Length (a, mm)	Single-Yarn Pull-Out Force (F, N)	Single-Yarn Displacement (δ, mm)	Single-Yarn Fracture Toughness (G_pf_, J/m^2^)	Two-Yarn Pull-Out Force (F, N)	Two-Yarn Displacement (δ, mm)	Two-Yarn Fracture Toughness (G_pf_, J/m^2^)	Three-Yarn Pull-Out Force (F, N)	Three-Yarn Displacement (δ, mm)	Three-Yarn Fracture Toughness (G_pf_, J/m^2^)
gCPO	20	22.15	30.28	670.70	43.50	27.91	1214.90	69.14	25.59	1769.29
	40	18.66	50.28	469.11	40.54	47.91	971.14	73.45	45.59	1674.29
	60	12.35	70.28	289.32	32.35	67.91	732.30	67.13	65.59	1467.69
	80	9.93	90.28	224.12	13.82	87.91	303.73	12.88	85.59	275.60
	100	7.11	110.28	156.82	9.26	107.91	199.85	11.94	105.59	252.15
	120	4.02	130.28	87.29	8.05	127.91	171.61	11.27	125.59	235.90
	160	1.47	170.28	31,29	4.16	167.91	87.31	4.69	165.59	97.08
KPO	20	15.43	26.49	408.74	52.10	27.11	1412.43	134.94	28.14	3797.21
	40	10.87	46.49	252.67	34.64	47.11	815.95	86.47	48.14	2081.33
	60	9.80	66.49	217.20	29.40	67.11	657.68	68.61	68.14	1558.36
	80	7.38	86.49	159.57	22.69	87.11	494.13	50.22	88.14	1106.60
	100	5.77	106.49	122.89	15.31	107.11	327.97	40.94	108.14	885.45
	120	4.70	126.49	99.08	13.55	127.11	287.06	26.72	128.14	570.65
	160	2.28	166.49	47.45	5.10	167.11	106.53	11.94	168.14	250.95

**Table 6:** Coefficients of static and kinetic friction results of CNT-grown p-aramid yarn structures in dry and wet states.

Label	Coefficent of Static Friction-Dry (μ_sc_)	Coefficent of Static Friction-Wet (μ_sc_)	Coefficent of Kinetic Friction-Dry (μ_kp_)
Single yarn			
gCPO	0.300 ± 0.01	0.300 ± 0.01	0.180 ± 0.12
KPO	0.220 ± 0.01	0.220 ± 0.01	0.150 ± 0.07
Multiple yarns			
Two yarns			
gCPO	0.320 ± 0.01	0.305 ± 0.01	0.150 ± 0.11
KPO	0.230 ± 0.01	0.223 ± 0.01	0.130 ± 0.04
Three yarns			
gCPO	0.320 ± 0.01	0.310 ± 0.01	0.160 ± 0.12
KPO	0.250 ± 0.01	0.240 ± 0.01	0.130 ± 0.03

## Data Availability

The raw data supporting the conclusions of this article will be made available by the authors on request.
